# Editorial: Use of chemosensitization to augment efficacy of antifungal agents, Volume II

**DOI:** 10.3389/ffunb.2023.1275400

**Published:** 2023-08-31

**Authors:** Jong H. Kim, Olihile M. Sebolai, Vitaly Dzhavakhiya

**Affiliations:** ^1^ Foodborne Toxin Detection and Prevention Research Unit, Western Regional Research Center, Agricultural Research Service, United States Department of Agriculture, Albany, CA, United States; ^2^ Department of Microbiology and Biochemistry, University of the Free State, Bloemfontein, South Africa; ^3^ Department of Molecular Biology, All-Russian Research Institute of Phytopathology, Moscow, Russia

**Keywords:** antifungal, azoles, azoxystrobin, chemosensitization, drug resistance, fungicide resistance, heat sensitization, natural products

Current antifungal interventions exhibit limited efficacy. There are three major issues with conventional antifungal drugs and fungicides. The first problem is the tendency of treated fungi to develop resistance to antifungal agents. For instance, there have been increasing numbers of yeast infections caused by non-*albicans* species, such as *Candida glabrata*, *Candida krusei* and *Candida auris*, with a higher frequency of drug resistance, including fluconazole resistance (Centers for Disease Control and Prevention (U.S.), 2023a; Centers for Disease Control and Prevention (U.S.), 2023b). Secondly, there is a potential for negative side-effects related to the use of conventional antifungal agents to the environment or the patient; for example, the polyene drug amphotericin B (AMB) triggered toxicity to the patients including nephrotoxicity; to avoid host toxicity, various formulations, such as lipid-associated AMB formulation have been developed (Hamill, 2013; Marena et al., 2022). A third, and surfacing problem, is stagnation in the development of new, effective antifungal agents; azole and polyene drugs were introduced to the clinical settings before 1980, while the echinocandin caspofungin was approved for clinical use after 2000, respectively (Houšť et al., 2020).

Infection of crops by fungal pathogens, including those that produce mycotoxins such as aflatoxins, fumonisins, ochratoxins, etc., is also problematic since effective fungicides for preventing fungi, especially fungicide-resistant pathogens, are often limited. Of note, over 25% of current fungicide sales are azoles, such as tebuconazole, metconazole, etc. (Bowyer and Denning, 2014; Huang et al., 2022). However, increased application of azoles could provide environmental selection pressure for the emergence of human pathogens, e.g., *Aspergillus fumigatus*, resistant to azole clinical drugs (voriconazole, itraconazole, posaconazole, etc.) (Centers for Disease Control and Prevention (U.S.), 2019), and for the accumulation of azole-resistant plant pathogens (Pereira et al., 2020).

This Research Topic describes recent advances in the field of antifungal chemosensitization, which is a strategy where combined application of a conventional antifungal drug or fungicide with a second compound, termed chemosensitizer, increases the efficacy of the drug/fungicide co-applied (Campbell et al., 2012). The chemosensitizers are, by definition, safe compounds and, when co-applied with a commercial antifungal agent, can result in a synergistic antifungal interaction. Therefore, the approach can provide a safe measure of improving the efficacy of conventional antifungal agents, resulting in lower dosages for antifungal treatment. The use of chemosensitizers also overcomes the resistance of fungal pathogens to commercial drugs or fungicides (Campbell et al., 2012).

In this Research Topic, four works (three original research articles, one review) were published on the recent advances in antifungal chemosensitization.

Species of *Scedosporium* and *Lomentospora* are opportunistic human pathogens causing invasive fungal infections. Since the sole use of azole drugs cannot effectively control these fungi, Wang et al. investigated the chemosensitization effect of the non-antifungal drugs tacrolimus (FK506; calcineurin inhibitor) and everolimus (mammalian target of rapamycin (mTOR) inhibitor) in combination with azole drugs against fifteen clinical fungal isolates. Synergistic effects were determined in the combined treatments of tacrolimus with itraconazole, voriconazole, and posaconazole on the test fungi, where the synergism was corresponded to the increased reactive oxygen species (ROS) activity (tacrolimus combined with itraconazole), early apoptosis (everolimus combined with itraconazole or voriconazole), and late apoptosis (tacrolimus combined with itraconazole or posaconazole); the synergism did not involve efflux pump activity. Therefore, the chemosensitization could broaden antifungal regimens to combat rare infections, such as *Scedosporium*/*Lomentospora* infections.

Dhandapani et al. provided a review of the recent antifungal chemosensitization strategies: (1) chemosensitization considerably enhanced the activity of conventional drugs by properly targeting cellular systems, making the fungal pathogens highly susceptible to commercial drugs co-applied, and (2) chemosensitizers could also address the mechanisms of drug resistance, thus combat the resistance of pathogens to antifungal drugs. Of note, natural compounds such as plant compounds or microbial proteins function as potent chemosensitizers to overcome the resistance in mycoses. For instance, the filamentous fungus *Aspergillus giganteus* produces a cysteine-rich extracellular protein termed antifungal protein (AFP), which enhanced antifungal efficacy against selected filamentous and/or non-filamentous fungal pathogens; AFP would serve as a potent chemosensitizer to augment the fungicidal efficacy of commercial antifungal drugs.

*Parastagonospora nodorum* is a necrotrophic pathogen that causes glume and leaf blotch of wheat, resulting in serious losses in grain yield. While the conventional fungicidal formulations to treat the pathogen are based mainly on triazoles and/or on triazoles combined with strobilurin fungicides, the prolonged application of these fungicides could result in the selection of fungicide-resistant strains of *P. nodorum* in the fields. In the study by Kartashov et al., chemosensitization was investigated for effective control of *P. nodorum* strains, including those resistant to tebuconazole or azoxystrobin. In the study, 6-demethylmevinolin (6-DMM), a metabolite of *Penicillium citrinum*, was co-applied as a chemosensitizer. The study identified that co-application of 6-DMM with tebuconazole not only augmented fungicidal effectiveness but also attenuated the fungal resistance to tebuconazole; a synergistic effect was observed in both preventive and post-inoculation treatments. Therefore, 6-DMM could be a putative component for new formulations with triazole and strobilurin fungicides, which improves fungicide efficacy and lowers the level of fungicides required for the effective control of pathogens.

Finally, Kim et al. reported the new utility of long-chain alkyl gallates as heat-sensitizing agents, enabling high-efficiency fungal pathogen intervention towards seed protection. Although heat treatment is one of the intervention strategies for fungal pathogen control in agricultural or food production, heat treatment can negatively affect the quality of the crop seeds or food products, including seed viability, nutritional value, texture, etc. The study identified that long-chain alkyl gallates, including octyl gallates (OG (octyl 3,4,5-trihydroxybenzoic acid)), can function as heat-sensitizing agents, thus lowering the temperatures necessary for effective seed sanitation; the heat-sensitizing capacity was unique to the long-chain alkyl gallates, where OG exhibited the highest activity, followed by decyl- and nonyl gallate. Therefore, OG-mediated heat sensitization would achieve safe, rapid, and cost-effective pathogen control in agriculture/food industry settings.

In summary, the research articles and review paper presented in this Research Topic provide useful information and recent progress on antifungal chemosensitization for fungal pathogen control. Identification of new, safe chemosensitizing molecules and cellular targets, as well as elucidation of their precise mechanisms of action, will further the effective control of fungal pathogens.

## Author contributions

JK: Conceptualization, Writing – original draft, Writing – review & editing. OS: Writing – review & editing. VD: Writing – review & editing.
